# Testing Cellular Proliferation Responses to Oxidative, Glucocorticoid and Metabolic Challenges in the Baboon

**DOI:** 10.1093/geroni/igaa057.3283

**Published:** 2020-12-16

**Authors:** Daniel Adekunbi, Cun Li, Peter Nathanielsz, Adam Salmon

**Affiliations:** 1 University of Wyoming, San Antonio, Texas, United States; 2 University of Wyoming, Laramie, Wyoming, United States; 3 University of Texas Health at San Antonio, San Antonio, Texas, United States

## Abstract

Aging is associated with progressive loss of cellular homeostasis which results from intrinsic and extrinsic challenges. Testing how such challenges relate to the aging process is often limited by the available model systems. We use primary cells (fibroblasts) isolated from baboons as a non-human primate cellular model to address how stressful challenges affect resilience. Using a real-time live-cell imaging system, we characterized a protocol for testing the effects of pro-oxidant compounds (e.g hydrogen peroxide (H2O2), paraquat and thapsigargin), dexamethasone and low glucose environment on cellular proliferation in fibroblasts derived from baboons across the life-course. The inhibitory effect of H2O2 (50 and 100µM), an oxidative stress, on cell proliferation was dose-dependent, with a higher impact in old males (age 14.52-14.80 years; average life span 21 years) compared to young males (6.35-6.39 years). Exposure to a different oxidative stress, paraquat (100 and 200µM) tended to reduce cell proliferation rate with age in males but not females. Inhibitory effects of thapsigargin, an endoplasmic reticulum stress inducer, on cell proliferation were dependent on challenge duration (2 vs 24h), concentration (0.1 and 1µM) and donor age, with greater resilience in young males than young females (4.33-6.70 years). Dexamethasone (100 and 500µM), a glucocorticoid, reduced cell proliferation dose-dependently, with older males exhibiting more resilience than females. In response to low glucose (1mM), cell proliferation reduced with age. Donor’s chronological age and sex are important variables in cellular response to challenge compounds faced during aging, which will guide our on-going studies on the cellular transcriptome and proteome.

